# Progressive pseudorheumatoid dysplasia with new-found gene mutation of Wntl inducible signaling pathway protein 3

**DOI:** 10.1186/s12969-018-0272-7

**Published:** 2018-09-10

**Authors:** Wenji Chen, Shiyan Mo, Gui Luo, Yanyan Wang, Xiaohu Deng, Jian Zhu, Wei Zhao

**Affiliations:** Department of Rheumatology, Hainan Branch of Chinese People’s Liberation Army General Hospital, Haitang Bay, Sanya, China

**Keywords:** Progressive pseudorheumatoid dysplasia, Wntl inducible signaling pathway protein 3

## Abstract

**Background:**

As one kind of osteochondrodysplasia, progressive pseudorheumatoid dysplasia (PPD) is also known as spondyloepiphyseal dysplasia tarda with progressive arthropathy or arthropathy progressive pseudorheumatoid of childhood. PPD is a very rare disease, especially in China, and has an estimated prevalence of 1/1000000 due to lacking definite prevalence survey. It is an autosomal recessive disorder caused by gene mutation of Wntl inducible signaling pathway protein 3 (WISP3). Its basic pathological change is persistent degeneration and loss of articular cartilage in multiple joints. Its clinical appearances include bone enlargement, platyspondyly, irregular endplate, secondary osteoarthritis, extensive osteoporosis, joint rigidity and function loss. Clinical diagnosis of PPD is made based on clinical appearance and imaging examinations, whereas its definite diagnosis depends on gene sequencing. PPD has no severe effect on life span, but causes high disability rate and very poor prognosis. There are only case reports with limited information in China.

**Case presentation:**

One female patient was diagnosed as PPD and secondary osteoarthritis. She had typical clinical appearance and imaging examinations, and received individualized therapeutic regimens. She had a gene mutation (*c.72delT, p.T24TfsX4*) of WISP3. This gene mutation has not been reported by previous literatures and included in Single Nucleotide Polymorphism Database.

**Conclusions:**

As the first time, this paper reported a patient with PPD caused by new-found gene mutation (*c.72delT, p.T24TfsX4*) of WISP3.

## Background

As one kind of osteochondrodysplasia, progressive pseudorheumatoid dysplasia (PPD) is also known as spondyloepiphyseal dysplasia tarda with progressive arthropathy (SEDTPA) or arthropathy progressive pseudorheumatoid of childhood (APPRC) [1]. It is an autosomal recessive disorder caused by gene mutation of Wntl inducible signaling pathway protein 3 (WISP3) [2]. Its basic pathological change is persistent degeneration and loss of articular cartilage in multiple joints. Patients with PPD have no abnormal appearance under 3 years of age, and most appearances appear between the ages of 3 and 8. As age increases, PPD aggravates gradually. Clinical diagnosis of PPD is made based on clinical appearances and imaging examinations, whereas its definite diagnosis depends on gene sequencing [3]. PPD is a very rare disease, especially in China, and has an estimated prevalence of 1/1000000 due to lacking definite prevalence survey. Since PPD has been described in 1986, there are only case reports with limited information in China [4]. Its clinical appearances include bone enlargement, platyspondyly, irregular endplate, secondary osteoarthritis, extensive osteoporosis, joint rigidity and function loss [5]. PPD has no severe effect on life span, but causes high disability rate and very poor prognosis [6]. As the first time, this paper reported a patient with PPD caused by new-found gene mutation of WISP3.

## Case presentation

This unmarried female was 20 years of age (Li minority). Main cause for admission is multiple joint swelling for 13 years, aggravated with pain for 5 years. When she was 7 years old, swelling appeared initially in bilateral knee joints, and then in bilateral metacarpophalangeal, interphalangeal, wrist, elbow and ankle joints. Bone enlargement appeared with pain, and ankle activity was severely limited when she was 15 years old. Due to poor economic condition and medical knowledge, her parents did not take her to see a doctor until she had bone enlargement and severely limited ankle activity. She has been diagnosed as rheumatoid arthritis, but her condition was not improved after the therapy of prednisone and methotrexate for more than 6 months. In recent 3 years, she had further aggravated swelling and pain of bilateral knee and elbow joints, and difficulties to walk surefootedly, straighten limbs and lift weights. She had no fever, rash, nausea, vomiting, morning stiffness, hair loss, dry mouth, dry eye, oral ulcer and Raynaud’s phenomenon. Her parents were health, and had no disease history.

No abnormality was found in chest and abdomen. There were bone enlargement, pain with press and limited activities in bilateral interphalangeal, wrist, elbow and knee joints. Bilateral wrist joints had limited activities of dorsal stretch (50°) and palmar flexion (50°). Bilateral elbow joints had limited activity of straighten (left elbow: 150°; right elbow: 160°). Patella grind test has positive result, and floating patella test has negative result. There was extensive anteflexion of lumbar vertebrae, but no limited activity in horizontal or vertical direction and pain with press in sacroiliac joints and paravertebral muscles. There were limited abilities of pronation and abduction in bilateral hip joints, and Patrick sign can not be performed by her.

Laboratory examinations: hemoglobin 113 g/L, white blood cell count 6.99 × 10^9^/L and platelet count 245 × 10^9^/L. Osteocalcin 36.92 ng/ml [normal reference values (females): 11-48 ng/ml], β-collagen degradation product 0.811 ng/ml [normal reference values (premenopausal females): 0.3–0.57 ng/ml] and total I type collagen amino terminal prolongation peptide 105μg/L [normal reference values (females): 19-84μg/L]. There were negative results of HLA-B27, rheumatoid factor, anti-cyclic citrullinated peptide and antinuclear antibody, and normal levels of immunoglobulins, complements and inflammatory markers.

**Fig. 1:**
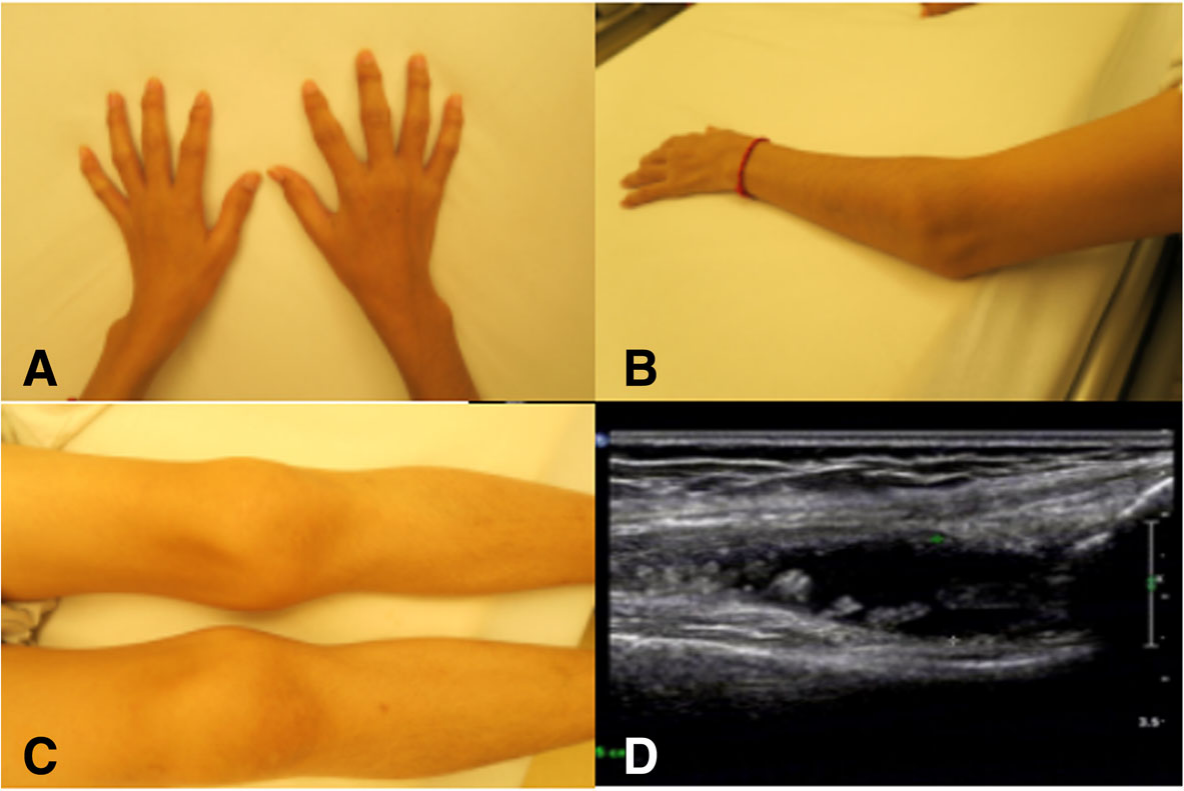
Pictures/ultrasound. Bone enlargement in multiple joints of hands (**a**); bone enlargement in elbow joint (**b**); bone enlargement in knee joints (**c**); ultrasound of knee joint: synovial hyperplasia and effusion in suprapatellar bursa, rough articular surface and inhomogeneous echo enhancement of hyaline cartilage (**d**)

**Fig. 2:**
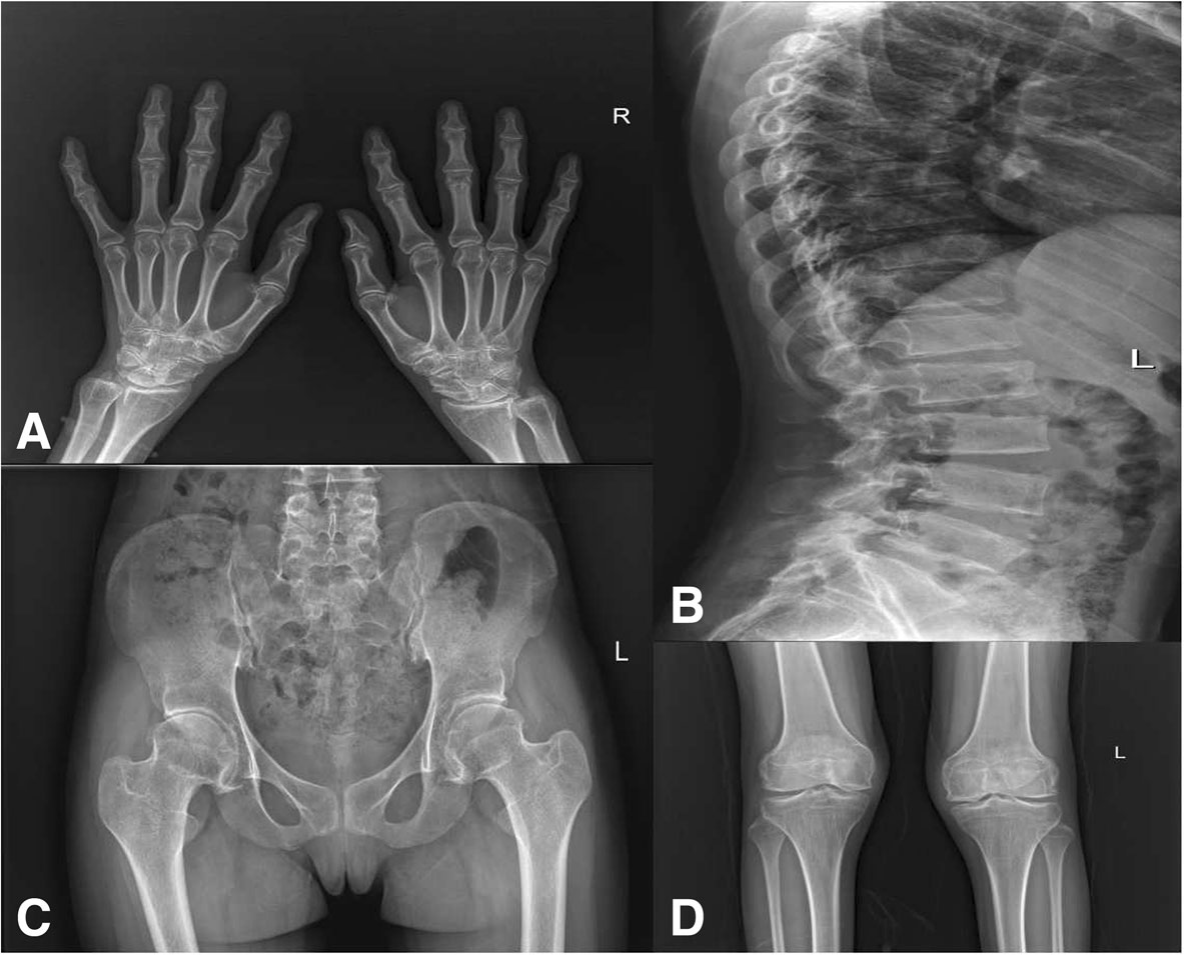
Plain films. Plain film of hands: osteoporosis (**a**); plain film of spine: thoracic and lumbar platyspondyly with irregular margin (**b**); plain film of pelvis: space narrowing of hip joint and normal sacroiliac joint (**c**); plain film of knee joints: joint space narrowing and rough articular surface (**d**)

Normal electrocardiography, echocardiography and chest computed tomography. Ultrasound of knee joint: 1) synovial hyperplasia and effusion in suprapatellar bursa; 2) rough articular surface; 3) inhomogeneous echo enhancement of hyaline cartilage (Fig. 1). Plain film of hands: osteoporosis. Plain film of spine: thoracic and lumbar platyspondyly with irregular margin. Plain film of pelvis: space narrowing of hip joint and normal sacroiliac joint. Plain film of knee joints: joint space narrowing and rough articular surface (Fig. 2). Bone mineral density: reduced bone substance. Bone scan: increased intake in multiple joints.

**Fig. 3:**
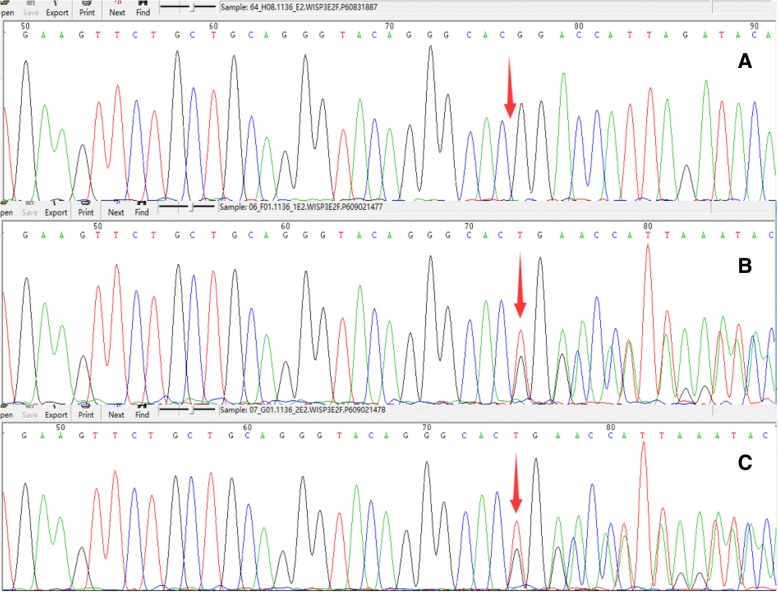
Gene sequencing. Gene sequencing results of Wntl inducible signaling pathway protein 3 in this patient (**a**) and her father (**b**) and mother (**c**)

Sanger was used to perform gene sequencing (Fig. 3). She had a gene mutation (*c.72delT, p.T24TfsX4*) of WISP3 (NM_003880.3), and her parents had its heterozygous gene mutation (*c.72delT, p.T24TfsX4*), in accordance with recessive inheritance pattern. Pathogenicity of mutation was predicted by sequencing experts. Frameshift mutation was caused and truncated protein was produced due to gene mutation. She was diagnosed as PPD and secondary osteoarthritis. Spondyloarthritis was excluded due to negative result of HLA-B27 and normal sacroiliac joint. Juvenile rheumatoid arthritis was excluded due to no damage of other organs, bone enlargement rather than bone destruction, negative results of rheumatoid factor, anti-cyclic citrullinated peptide and antinuclear antibody, and normal levels of immunoglobulins and complements. Overlap arthritis was excluded due to negative results of rheumatoid factor, anti-cyclic citrullinated peptide and antinuclear antibody, and normal levels of immunoglobulins and complements. She received the following therapeutic regimens: 1, symptomatic treatment: physical therapy and pain management; 2, glucosamine sulfate and diacerein; 3, psychological counseling; 4, regular reexamination. At present, her pain was controlled and limited activity in joints was improved after continuous treatment.

## Discussion

Ruth Wynne-Davies has reported PPD in 1982 as the first time, and realized that PPD is an autosomal recessive disorder [7]. Jennifer R. Hurvitz has firstly found that PPD derives from gene mutation of WISP3 in 1999 [2]. Several gene mutation of WISP3 have been reported all over the world, such as deletion, replacement, insertion and missense, and there were also some findings in China [4]. As the first time, this paper found a new gene mutation (*c.72delT, p.T24TfsX4*), which has not been reported by previous literatures and included in Single Nucleotide Polymorphism Database. This gene mutation may lead to high PPD risk through promoting frameshift mutation and producing truncated protein in the process of protein translation.

Also known as connective tissue growth factor CCN6 gene, WISP3 gene has been firstly cloned and located at 6q 22-6q 23 [2]. Gene mutation of WISP3 leads to PPD development through still unclear mechanisms. As a secretory protein consisting with signal peptide and 4 structural domains, gene mutation of WISP3 may inhibit its extracellular secretion, cause its intracellular accumulation and interfere its normal function. WISP3 may maintain the stability of cartilage through regulating the synthesis of type II collagen and protein polysaccharide, whereas gene mutation of WISP3 may cause the lack of stability and loss of cartilage [8]. WISP3 may inhibit the hyperplasia of cartilage and promote the differentiation of cartilage, whereas gene mutation of WISP3 may accelerate extensive hyperplasia and early degeneration of cartilage, which leads to joint deformation and PPD development [9].

Clinical features of PPD are progressive and symmetrical swelling and deformation of multiple joints. As age increases, joints deform and pain aggravates gradually [3]. Patients with PPD have joint rigidity, limited activity and short stature, which severely affect their labor capacity and life quality. Patients with PPD also have spinal lesion, but no clinical appearance at early stage. Patients older than 15 years begin to have lumbar lordosis, thoracic kyphosis, spinal scoliosis and bow-backed deformation [10]. Not only thickening of ligamentum flavum and posterior longitudinal ligament, but also protrusion of intervertebral disc lead to spinal canal stenosis and spinal nerve compression. Imaging examinations show that there are bone enlargement, space narrowing, platyspondyly, irregular endplate, secondary osteoarthritis and extensive osteoporosis around multiple joints in patients with PPD. As described above, this paper reported a PPD patient with typical clinical appearance and imaging examinations.

PPD has no etiological treatment available now, and individualized therapeutic regimens may be beneficial to patients with PPD. [11] Symptomatic treatment, such as physical therapy and pain management, can improve subjective feeling and life quality of patients with PPD. Because basic pathological change of PPD is persistent degeneration and loss of articular cartilage, glucosamine sulfate and diacerein were tentatively applied by us with still unclear therapeutic effects [12]. Psychological counseling can alleviate the psychological burden of patients cause by PPD, and help them understand this disease and avoid unnecessary treatment. In order to avoid PPD development in next generation, prospective spouses should receive genetic examinations before marriage. Surgical operation, such as osteotomy and arthroplasty, can be applied for PPD patients at advanced stage with hip and knee joints severely involved.

## Conclusions

As the first time, this paper reported a patient with PPD caused by new-found gene mutation of WISP3 (*c.72delT, p.T24TfsX4*).

## Abbreviations

APPRC: Arthropathy progressive pseudorheumatoid of childhood
PPD: Progressive pseudorheumatoid dysplasia
SEDTPA: Spondyloepiphyseal dysplasia tarda with progressive arthropathy
WISP3: Wntl inducible signaling pathway protein 3

## References

[1] Madhuri V, Santhanam M, Rajagopal K, Sugumar LK, Balaji V (2016). Bone Joint Res.

[2] Hurvitz JR, Suwairi WM, Van Hul W, El-Shanti H, Superti-Furga A, Roudier J (1999). Mutations in the CCN gene family member WISP3 cause progressive pseudorheumatoid dysplasia. Nat Genet.

[3] Garcia Segarra N, Mittaz L, Campos-Xavier AB, Bartels CF, Tuysuz B, Alanay Y (2012). The diagnostic challenge of progressive pseudorheumatoid dysplasia (PPRD): a review of clinical features, radiographic features, and WISP3 mutations in 63 affected individuals. Am J Med Genet C Semin Med Genet.

[4] Yan W, Dai J, Xu Z, Shi D, Chen D, Xu X (2016). Novel <i>WISP3</i> mutations causing progressive pseudorheumatoid dysplasia in two Chinese families. Hum Genome Var.

[5] Rai E, Mahajan A, Kumar P, Angural A, Dhar MK, Razdan S (2016). Whole Exome Screening Identifies Novel and Recurrent WISP3 Mutations Causing Progressive Pseudorheumatoid Dysplasia in Jammu and Kashmir-India. Sci Rep.

[6] Wickrematilake G (2017). Progressive Pseudorheumatoid Dysplasia or JIA?. Case Rep Rheumatol.

[7] Wynne-Davies R, Hall C, Ansell BM (1982). Spondylo-epiphyseal dysplasia tarda with progressive arthropathy. J Bone Joint Surg Br.

[8] Sen M, Cheng YH, Goldring MB, Lotz MK, Carson DA (2004). WISP3 dependent regulation of type II collagen and aggrecan production I chondrocytes. Arthritis Rheum.

[9] Zhou HD, Bu YH, Peng YQ, Xie H, Wang M, Yuan LQ (2007). Cellular and molecular responses in progressive pseudorheumatoid dysplasia articular cartilage associated with compound hetemzygous WISP3 gene mutation. J Mol Med.

[10] Montané LS, Marín OR, Rivera-Pedroza CI, Vallespín E, Del Pozo Á, Heath KE (2016). Early severe scoliosis in a patient with atypical progressive pseudorheumatoid dysplasia (PPD): Identification of two WISP3 mutations, one previously unreported. Am J Med Genet A.

[11] Hartmann M, Merker J, Haefner R, Haas JP, Schwirtz A (2016). Biomechanics of walking in adolescents with progressive pseudorheumatoid arthropathy of childhood leads to physical activity recommendations as therapeutic focus. Clin Biomech.

[12] Gao YS, Ding H, Zhang CQ (2013). Total hip arthroplasty in a 17-year-old girl with progressive pseudorheumatoid dysplasia. J Clin Rheumatol.

